# Generation of a sexually mature individual of the Eastern dwarf tree frog, *Litoria fallax*, from cryopreserved testicular macerates: proof of capacity of cryopreserved sperm derived offspring to complete development

**DOI:** 10.1093/conphys/coy043

**Published:** 2018-08-15

**Authors:** Rose Upton, Simon Clulow, Michael J Mahony, John Clulow

**Affiliations:** 1 School of Environmental and Life Sciences, University of Newcastle, Callaghan, NSW 2308, Australia; 2 Department of Biological Sciences, Macquarie University, Sydney, NSW 2109, Australia

**Keywords:** ART, assisted reproductive technologies, conservation, cryopreservation, IVF, sperm

## Abstract

Amphibians are the most threatened vertebrate class globally based on recent rates of decline and extinction. Sperm cryopreservation and other assisted reproductive technologies have the potential to help manage small and threatened populations and prevent extinctions. There are a growing number of reports of recovery of amphibian sperm after cryopreservation, but relatively few published reports of amphibian embryos generated from frozen sperm developing beyond metamorphosis to the adult stage and achieving sexual maturation. In this study on the Eastern dwarf tree frog (*Litoria fallax*), a temperate amphibian species from eastern Australia, a small number of viable metamorphs and one sexually mature male frog (itself producing sperm) were produced from cryopreserved sperm, demonstrating the capacity of embryos generated from cryopreserved sperm to complete the life cycle to sexual maturity. Low progression rates between developmental stages were not deemed to be due to effects of cryopreservation, since control embryos from unfrozen sperm had a similarly low progression rate through development.

## Teaser

There are limited studies reporting the use of cryopreserved sperm in amphibians, and fewer reporting the generation of sexually mature individuals. This paper gives proof of concept to the value of genome banking for amphibian conservation by demonstrating in one amphibian species, the Eastern dwarf tree frog (*Litoria fallax*), that offspring produced from cryopreserved sperm are capable of achieving metamorphosis and reaching sexually maturity.

## Introduction

Assisted reproductive technologies (ARTs) for amphibians are of great interest because of their potential to assist the conservation of threatened amphibians that now face decline and extinction at a rate that is unparalleled amongst the vertebrates (Clulow *et al.*, 2014; Clulow and Clulow, 2016; Bower *et al.*, 2017). There are a large number of species requiring direct intervention to prevent extinction through captive breeding, but this can be costly and leads to problems of inbreeding and loss of genetic diversity (Snyder *et al.*, 1996). The use of sperm cryopreservation to reduce captive colony costs by reducing the number of live animals required to maintain genetic diversity increases the efficiency and cost-effectiveness of such programmes (Clulow *et al.*, 2014).

Considerable progress in cryopreservation of amphibian sperm has been reported since the first reports of the generation of embryos from cryopreserved sperm in 1998 (Browne *et al.*, 1998; Mansour *et al.*, 2009; Shishova *et al.*, 2011; Langhorne *et al.*, 2013; Uteshev *et al.*, 2013; Clulow *et al.*, 2014; Clulow and Clulow, 2016; Pearl *et al.*, 2017). Nevertheless, most reports of amphibian sperm cryopreservation deal with the recovery of sperm assessed by parameters such as motility and vitality but do not report developmental success to metamorphosis or beyond.

There are only a small number of studies that report development from cryopreserved sperm to blastulae (Browne *et al.*, 1998), tailbud stage (Proaño and Pérez, 2017) swimming larvae (Mansour *et al.*, 2009; Shishova *et al.*, 2011; Langhorne *et al.*, 2013; Uteshev *et al.*, 2013), one review reporting unpublished results of development past metamorphosis (Browne *et al.*, 2011), and only one study that demonstrates the complete life cycle of amphibians generated from frozen sperm (Pearl *et al.*, 2017).

The production of sexually mature adults from cryopreserved sperm is an important proof of concept in assisted reproduction generally, but especially for amphibians, since amphibian larvae produced by procedures such as nuclear transfer do not necessarily reach metamorphosis or develop to maturity (Di Berardino *et al.*, 2003). Hence there is a need to demonstrate the completion of development to metamorphosis and beyond to the adult stage and sexual maturity in amphibians generated with cryopreserved sperm across a range of species to establish general applicability of the approach.

Here we report the completion of development to metamorphosis and sexual maturity of offspring produced from cryopreserved sperm of the Eastern dwarf tree frog.

## Materials and methods

*Litoria fallax* is a pond-breeding species that spawns in a range of habitats across several months of the austral spring and summer (September to April), although males may call throughout the year. Thirty-five calling *L. fallax* males and seven gravid, ovulated females (found in amplexus following rain and storm events) were collected from ponds located in the Hunter Estuary (−32.84 S, 151.69 E), under NSW Scientific Licence SL101269, and euthanized by immersion in 0.4% w/v ethyl 3-aminobenzoate methanesulfonate (MS-222; Sigma Aldrich; E10521) buffered with 0.4% w/v sodium bicarbonate, followed by excision of the heart.

Males were collected ahead of time over December 2015 and housed in plastic terraria (30 cm × 18 cm × 20 cm) containing autoclaved gravel and aged tap water (to create an environment ~25% terrestrial and 75% aquatic) with no more than 15 individuals to a tank. Terraria were exposed to natural day light cycles and were supplied with crickets supplemented with Multical calcium/vitamin powder (Vetafarm, Wagga Wagga, NSW) three times weekly. Individuals were kept in this manner for at least a week prior to experiments taking place. Females found in amplexus on the 30 December 2015 were separated from the amplecting male and housed at 10°C in controlled temperature cabinets (TRISL-1175-2-SD; Thermoline Scientific, Wetherill Park, NSW) to delay oviposition (Sherman *et al.*, 2010), allowing a delay in the commencement of IVF trials until 10–15 h following collection of animals from the field. All experimental work involving animal use was completed in accordance with the necessary guidelines under University of Newcastle ethics approval A-2013-328.

Four testicular macerates were prepared in 100 μl Simplified Amphibian Ringer (SAR; 113mM NaCl, 1mM CaCl_2_, 2mM KCl, 3.6mM NaHCO_3_; recipe as used by Browne *et al.*, 1998) by teasing apart the testes within the specified volume using fine forceps. One of these was used as an unfrozen control macerate (UF1), used purely to demonstrate viability and fertility of oocytes, and pooled both testes from five male *L. fallax*. The remaining three macerates (CR1, CR2 and CR3) each pooled both testes of 10 male *L. fallax* per macerate (to adjust for loss of sperm and a decrease in sperm concentration following cryopreservation and washing procedure), and were cryopreserved in the following way: each macerate was extended at a dilution ratio of 1:6 with 15% v/v dimethyl sulfoxide (Me_2_SO) and 1% w/v sucrose solution immediately after maceration, before being loaded into 250 μl cassou straws. The cryoprotectant solution was modified from that of Browne *et al.* (1998), following results of experimental observations indicating that 1% w/v sucrose was more appropriate for this species than 10% w/v sucrose in the extender (our unpublished work). Straws were loaded into the chamber of a controlled programmable freezing device (Cryologic, model CL3300) and frozen using a cooling ramp adapted from Browne *et al.* (1998); briefly, this included a 10 min hold step at 2° followed by a −1°C min^−1^ ramp to −8°C, a −3°C min^−1^ ramp to −16°C and a −3.4°C min^−1^ ramp to −80°C, followed by quenching in liquid nitrogen (Browne *et al.*, 1998).

Straws were subsequently thawed at room temperature (after a minimum quench time of 30 min within liquid nitrogen) and taken through a wash protocol to remove cryoprotectants. Thawed macerates were centrifuged twice for 2 min at 1000× *g*, first in 500 μl, and a second time in 100 μl of SAR. Following centrifugation, each macerate was activated by a 1:6 dilution in distilled water and added to ova (removed from the oviducts of euthanized, ovulated females) in a dry 3.5 mm diameter petri dish. Twenty minutes was allowed for fertilization (indicated by rotation of the animal pole to the upper surface of the ovum) before flooding the dish with 2 ml of 10% v/v SAR.

The ova from seven female *L. fallax* (found in amplexus) were used in the experiment, with 30–100 oocytes per petri dish depending on the number of ova recovered from each female. Ova from each female were fertilized with unfrozen macerate UF1 and with two of CR1, CR2 or CR3 frozen–thawed macerates. Sperm concentrations were not measured directly in individual dishes but were calculated from the macerates after cryopreservation and were in the order of 2.75 × 10^6^ for the fresh macerate and 4.2 × 10^5^ to 2.5 × 10^6^ to cells per ml for two of the cryopreserved macerates, but were not measured in the third.

After the completion of IVF, the hatching, development and metamorphosis of the fresh control and experimental cryopreserved sperm treatments were monitored for 20 weeks. The sexual maturity of two adult (both male) frogs derived from the IVF procedures (observed ~1 year after fertilization), one resulting from the use of thawed, cryopreserved sperm and one resulting from the control group using unfrozen sperm, were tested by determining the presence of sperm in urine after administration of human chorionic gonadotropin (hCG) (Chorulon; MSD Animal Health, Bendigo, Victoria) at a dosage rate of 3 IU/g body weight (a dosage rate within reported range causing sperm release in anurans (Kouba *et al.*, 2012; Clulow *et al.*, 2018)).

Frequency data were analysed by chi-square contingency analysis, subjected to Yates correction where cell numbers were below 10. Statistical calculations were computed via the *Vassarstats* portal (http://www.vassarstats.net/).

## Results

**Figure 1:**
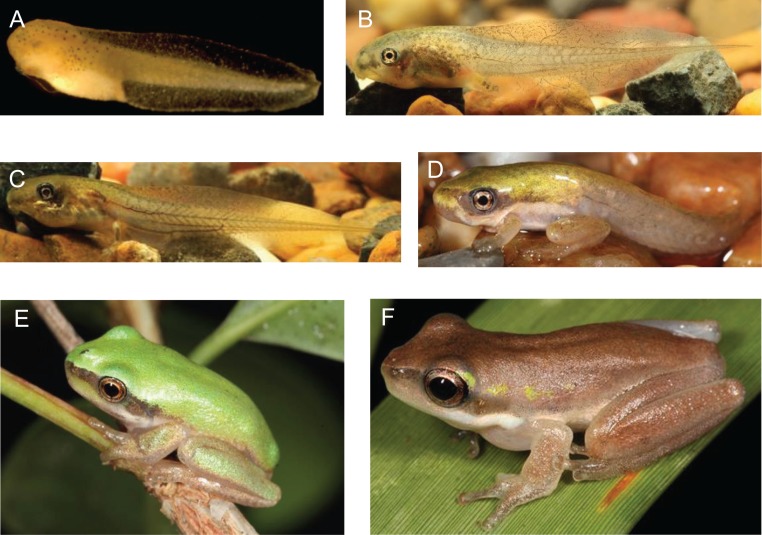
Images of *L. fallax* of varying developmental stages produced from cryopreserved testicular macerates in this investigation (Gosner Stages 20–46). **A**. freshly hatched, free-swimming tadpole; 5 mm, Gosner Stage 20. **B**. Tadpole nearing metamorphosis, hind legs beginning to develop; Gosner Stage 35, 20–30 mm. **C**. Tadpole with fully developed hind legs; Gosner Stage 40, 30–40 mm. **D**. Metamorph, Forelimb eruption and tail resorption commenced; Gosner Stage 42, 30–40 mm. **E**. Juvenile Frog; Tail resorbed; Gosner Stage 46, 10–20 mm. **F**. Sexually mature male, 20–30 mm. Photo credit: R. Upton (A), S. Mahony (B–F).

The number of embryos produced from each treatment (summed across the replicates) and the progression to later developmental stages are shown (Table 1). Progression to hatched tadpoles was significantly higher in cryopreserved replicates (*Χ*^2^, *P* < 0.001), and progression to mature tadpoles (Fig 1B–C) from hatched tadpoles (Fig. 1A) was nominally higher (but not statistically higher with Yates corrected *Χ*^2^, *P* = 0.086) in unfrozen macerate IVF’s.

**Table 1:** Development to various life cycle stages from ova of *L. fallax* fertilized with unfrozen control (*n* = 7 IVF replicates) and cryopreserved testicular macerates (*n* = 14 IVF replicates)

Stage	Unfrozen macerates—no. surviving (7 IVF replicates)	Cryopreserved macerates—no. surviving (14 IVF replicates)
Embryo (Gosner Stages 1–19)	351/413 (84.9%)	216/603 (35.8%)
Hatched Tadpole (Gosner Stage 20)	105 (29.9%)	133 (61.5%)
Mature Tadpoles (pre-metamorphosis; Gosner stage 41)	10 (9.5%)	4 (3.0%)
Metamorphosis (undergoing metamorphosis; Gosner stages 42–45)	1	4
Juvenile Frog (tail absorbed; Gosner Stage 46)	1	3
Sexually Mature Frog	1	1

Percentage values indicate percent at previous stage proceeding to the following stage in each treatment.

Taken together, the data show a similar progression between unfrozen and cryopreserved macerate offspring, indicating that the relatively low progression to metamorphosis and sexual maturity (Fig. 1) was a function of loss between developmental stages and not due to deleterious effects of macerate cryopreservation.

## Discussion

The purpose of this study was to demonstrate that cryopreserved sperm of *L. fallax* are capable of producing viable larvae that reach metamorphosis and sexual maturity. Although, no adult females were produced in this study, and the overall survival rates were low, the production of sperm from the male generated from cryopreserved sperm indicates that sexual maturity and the production of gametes in offspring generated from frozen sperm in this species is feasible. Low survival rates observed during the study were most likely due to sub-optimal IVF conditions or ova quality that impacted later development stages, resulting in few individuals reaching metamorphosis and beyond. Nevertheless, as embryos experienced similar rates of abnormalities within the unfrozen treatment compared to the cryopreserved sperm treatment, the cause of the problems are not related to cryopreservation. It is likely that further optimization of the IVF protocol used here may result in a higher hatching rate from both unfrozen and cryopreserved sperm IVF in this species. Further investigation should result in improved developmental success after IVF in this species. IVF conditions that need to be investigated to increase embryo survival include holding times for ova and sperm prior to mixing, ionic composition and osmolality of media (Edwards *et al.*, 2004) and possibly inclusion of anti-oxidants, since reactive oxygen species damage to gametes and embryos is a possible cause of embryonic loss (Guerin *et al.*, 2001).

Even though there are reports of the cryopreservation of sperm from a growing number of amphibian species, and the generation of embryos and tadpoles for some of those species (Browne *et al.*, 1998; Mansour *et al.*, 2009, 2010; Shishova *et al.*, 2011; Langhorne *et al.*, 2013; Uteshev *et al.*, 2013; Clulow *et al.*, 2014; Clulow and Clulow, 2016; Pearl *et al.*, 2017; Proaño and Pérez, 2017), this study provides one of only two reports for amphibians in which the production of sexually mature adult frogs derived from cryopreserved sperm has been demonstrated, either through the production of a mature individual producing gametes (as in this study), or through the generation of fertile adults that subsequently reproduced (Pearl *et al.*, 2017). Consequently, this study provides additional proof of concept support for the capacity to use ARTs in declining amphibian species in captive breeding and conservation programmes. Nevertheless, the authors know of only one captive breeding programme involving an endangered species that has incorporated the use of cryopreserved sperm to add back lost wild-type genes to the captive bred population (Howard *et al.*, 2015).

Given the serious decline of amphibian species globally, there is pressure to apply efficient and optimized captive breeding to amphibian conservation using best practice approaches that avoid adverse outcomes such as selection for domestic traits and loss of genetic fitness associated with captive breeding (Snyder *et al.*, 1996; Kraaijeveld-Smit *et al.*, 2006; Griffiths and Pavajeau, 2008; Bowkett, 2009). There is a strong argument that the proof of concept of capacity to generate sexually mature animals from cryopreserved sperm demonstrated here and elsewhere should be considered by amphibian conservation programme managers for incorporation into their breeding protocols to maximize retention of wild-type genetic diversity.
